# Biocompatibility and Biological Corrosion Resistance of Ti–39Nb–6Zr+0.45Al Implant Alloy

**DOI:** 10.3390/jfb12010002

**Published:** 2020-12-29

**Authors:** Yu-Jin Hwang, Young-Sin Choi, Yun-Ho Hwang, Hyun-Wook Cho, Dong-Geun Lee

**Affiliations:** 1 Department of Materials Science and Engineering, Inha University, Incheon 22212, Korea; yjhwang94@naver.com; 2 Department of Materials Engineering, Hanyang University, Ansan 15588, Korea; emailwnth@kitech.re.kr; 3 Korea Institute of Industrial Technology (KITECH), Incheon 21999, Korea; 4 Department of Pharmarcy, Sunchon National University, Suncheon 57922, Korea; hyh7733@naver.com; 5 Department of Biology, Sunchon National University, Suncheon 57922, Korea; hwcho@sunchon.ac.kr; 6 Department of Materials Science and Metallurgical Engineering, Sunchon National University, Suncheon 57922, Korea

**Keywords:** Ti-39Nb-6Zr alloy, biocompatibility, biological corrosion resistance, mechanical property

## Abstract

Titanium and titanium alloys are promising implant metallic materials because of their high strengths, low elastic moduli, high corrosion resistances, and excellent biocompatibilities. A large difference in elastic modulus between the implant material and bone leads to a stress shielding effect, which increases the probability of implant separation or decrease in the bone density around it. Thus, a lower elastic modulus is required for a better implant metallic material. β titanium has a lower elastic modulus and high strength and can reduce the probability of the stress shielding effect. In this study, the applicability of the Ti–39Nb–6Zr+0.45Al alloy, obtained by adding a small amount of aluminum to the Ti–39Nb–6Zr alloy, as a biomedical implant material was evaluated. The mechanical properties and biocompatibility of the alloy were evaluated. The biocompatibility of Ti–39Nb–6Zr+0.45Al was similar to that of Ti–39Nb–6Zr according to in vitro and in vivo experiments. In addition, the biological corrosion resistances were evaluated through a corrosion test using a 0.9% NaCl solution, which is equivalent to physiological saline. The corrosion resistance was improved by the addition of Al. The yield strength of the Ti–39Nb–6Zr+0.45Al alloy was improved by approximately 20%. The excellent biocompatibility confirmed its feasibility for use as a biomedical implant material.

## 1. Introduction

Titanium and titanium alloys are promising biomaterials because of their excellent biocompatibilities, specific strengths, corrosion resistances, and low elastic moduli compared to other metallic materials [1,2,3,4]. β-titanium has a low elastic modulus, is slightly different from that of the human bone (10–30 GPa), and has superior notch fatigue strength and wear resistance compared to those of the α+β titanium alloy, which has attracted considerable interest [5,6,7,8]. In addition, although the commonly used Ti–6Al–4V has a low ionic elution level, alloys composed of biocompatible elements attract increasing attention because of the concerns about the toxicity of V [9,10].

It is necessary to satisfy the requirements of biocompatibility, elastic modulus, and strength for the use of metallic materials as implant materials. If the biocompatibility is not satisfactory, fibrous connective tissue membranes are formed around implants, which lead to numerous problems, including chronic inflammation, which is one of the causes of implant failure [11]. In addition, a larger difference in the elastic modulus between the bone and implant leads to a lower stress transfer to the bone and lower load-carrying capacity of the implant, which causes changes in tissues and leads to osteoporosis due to the reduced bone density and separation of the implant [12,13]. Finally, if the implant material does not satisfy the requirements of yield and fatigue strengths, the implant easily breaks, becomes less stable as a component material, and thus unsuitable for use as a biomedical material.

The Ti–39Nb–6Zr (TNZ40) alloy used for a comparative evaluation in this study is suitable for use as an implant material [14,15]. This alloy consists of elements harmless to the human body and has a lower elastic modulus, below 60 GPa. Owing to its low tensile and yield strengths, a small amount of the α stabilizing element, Al, was added to compensate for this disadvantage. The addition of Al as an alloying element in titanium improves the tensile strength, creep strength, and fracture toughness, but it increases the elastic modulus [16,17]. On the other hand, when the Al content exceeds about 7% in titanium alloy, intermetallic compounds such as Ti_3_Al may be formed, making the alloy brittle. In this study, the Ti–39Nb–6Zr+0.45Al (TNZA) alloy was analyzed and compared to the TNZ40 alloy to evaluate the mechanical and biological corrosion properties and biocompatibility. In addition, in vitro and in vivo experiments were carried out to assess the stabilities and biocompatibilities of the implant materials.

## 2. Materials and Methods

### 2.1. Specimen Preparation and Biological Corrosion Resistance Test

The material used in this experiment was a β-titanium alloy of Ti–39Nb–6Zr+0.45Al (wt.%). For the in vivo experiments, the specimens were wire-cut to dimensions of 4 (width) × 5 (length) × 1 (thickness) mm^3^. In addition, the specimens used in the in vitro and in vivo experiments were polished to #2000 to allow cells to adhere to the surfaces of the specimens. After manufacturing the specimen according to the ASTM E8M (American Society for Testing and Materials, West Conshohocken, PN, USA) subsize specification, the mechanical properties of the titanium cladding were evaluated three times in each condition at a tensile speed of 1.5 mm/min at room temperature using a universal testing machine (INSTRON 5882, USA).

The corrosion resistance was evaluated in a corrosive atmosphere similar to that of the human body. After stabilizing the specimen under open-potential conditions for 10 min in a 0.9% NaCl (pH = 7) solution, the normal saline solution, potential scanning was carried out at a speed of 1 mV/s. The potential measurement range was configured by *E*_L_ = 2 V (*E*_oc_) (vs. Ag/AgCl/saturated NaCl electrode) at *E*_i_ = −1 V (*E*_oc_). Three iterations were carried out to achieve reproducibility for each specimen in which the polar resistance was obtained using the Stern–Geary equation.

### 2.2. In Vitro Experiment

A cell proliferation experiment, one of the in vitro experiments, was carried out to determine the cellular suitability of the TNZA alloy. An osteosarcoma cell line maintained in liquid nitrogen, MG-63 cells, which were obtained from American Type Culture Collection (ATCC, Rockville, MD, USA) were used. First, 1 ml (1.2 × 10^5^ per mL) was dispensed on each specimen, which was then incubated in an incubator (37 °C, 4.5% CO_2_) for 72 h.

The cells incubated for 72 h were treated with 100 ml of trypsin-EDTA (C_10_H_16_N_2_O_8_) solution. The cells were retrieved after maintenance for 1 min in the incubator. The number of retrieved cells was measured using a blood counting chamber. In addition, the absorption was measured at 550 nm using a microplate reader.

### 2.3. In Vivo Experiment

**Figure 1:**
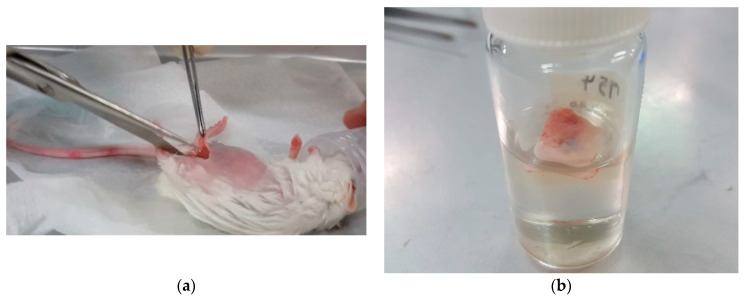
(**a**) Specimen insertion and (**b**) abdominal tissue fixed for more than 48 h in the 4% glutaraldehyde solution.

The animal experiment was conducted with the approval of the Institutional Animal Care and Use Committee at Sunchon National University (Approval No. SCNU IACUC-2016-03). Eight female Institute of Cancer Research (ICR) mice for the in vivo experiment, which were purchased from the Orientbio (Kwangju, South Korea), were divided into two groups of four mice and placed in a breeding box to adapt to the breeding room environment for one week. The temperature, relative humidity, and lighting cycle of the breeding room were controlled at 22 ± 2 °C, 50 ± 5%, and 12L:12D, respectively. The surfaces of the titanium specimens, which were implanted in the bodies of the mice, were disinfected with ethanol after ultrasonic cleaning. Then, the tools used in the experiment were wrapped in a gauze and sterilized in an autoclave for 1 h at 121 °C and 15 min at 1 atm. Ether was inhaled to anesthetize the mice to insert the TNZ40 and TNZA specimens. The abdominal hair of the mice was removed using a hair conditioner. The abdominal skin was incised to approximately 1 cm. The specimen was inserted into the subcutaneous tissue using a pincette and sealed using Duoderm (Extra-thin, ConvaTec, Cheshire, UK) (Figure 1a). After inserting the specimen, the mice were cultivated for four weeks, and then, the growing abdominal hair was removed again using a hair conditioner. The abdominal section where the specimen was inserted was cut off and placed and fixed in a 4% glutaraldehyde solution for at least 48 h (Figure 1b). The specimens were carefully removed from the abdominal tissue using a pincette, and then the tissues were refined for washing with water for 24 h with a 0.01-M phosphate buffer (pH = 7.4). Then, dehydration was carried out using 70% (twice), 80% (once), 95% (twice), and 100% (three times) alcohol at intervals of 1 h. The specimens were embedded and hardened using a JB-4 solution. The tissues were sliced to a thickness of 2.5 μm using a microsaw and placed on a slide glass to dry for 15 min using a 35 °C slide warmer. The sliced tissues were dyed using the hematoxylin–eosin method and sealed with Permount.

The sealed sliced tissues were observed using an optical microscope. The thickness of the newly formed fibrous connective membrane on the abdominal muscle tissue was measured at ten locations arbitrarily using Straight Line Length of the SPOT INSIGHT™ software (v4.0, Diagnostic Instruments, Sterling Heights, MI, USA). In addition, the rectangular area in the SPOT INSIGHT™ program was used to improve the reliability of the data measured for the membrane. The number of fibrous cells within an area of 5000 µm^2^ in the fibrous connective tissue formed on the section of the abdominal muscle tissue was measured. This method was implemented five times to obtain the average number of fiber cells. In addition, the number and length of the newly generated giant multi-nucleated cells (GMCs) were measured around the connective tissue in which the measurement of the number of GMCs was performed by considering the cells as a cluster. The difference between TNZ40 and TNZA was considered to be significant for *p* < 0.05 using a *t*-test.

## 3. Results and Discussion

### 3.1. Change in Yield Strength (YS)

**Figure 2:**
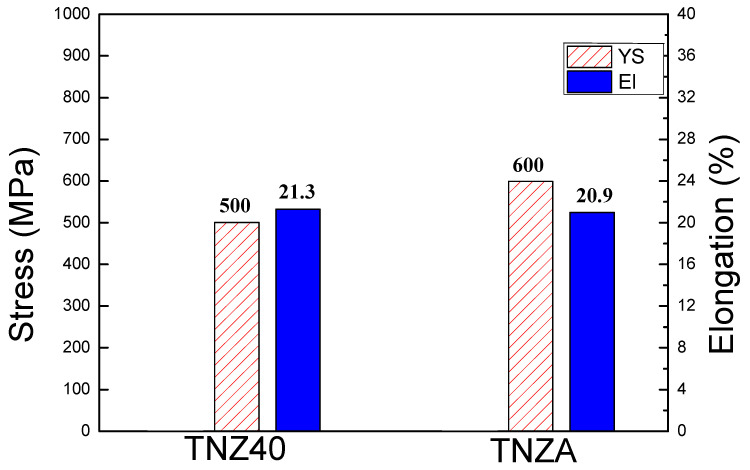
Tensile properties of the TNZA and TNZ40; yield strength and elongation.

The room-temperature tensile properties of the TNZA and TNZ40 alloys are shown in Figure 2. The YS of the TNZA alloy was approximately 600 MPa, while the elongation was approximately 20.9% and the elastic modulus was 59 GPa. The YS, elongation, and elastic modulus of the TNZ40 alloy were approximately 500 MPa, 21.3%, and 45 GPa respectively. Notably, the addition of a small amount of Al as an alloying element improved the YS by 20% (100 MPa), as Al is solid-solutioned as a substitutional atom in the matrix. The atomic sizes of Ti, Al, Zr, and Nb were 0.145, 0.143, 0.159, and 0.146 nm, respectively. As Al atoms are solid-solutioned within the Ti–39Nb–6Zr matrix, a misfit in the atomic lattice exists. The misfit between Ti and Zr is larger than that between Ti and Nb, owing to the Al element. The solid-solutioned atoms affect the friction stress in the dislocation movement, which increases the YS, limiting the dislocation movement [18,19]. The YS reflects the resistance to plastic deformation, which can be increased by adding Al. This improves the strength in the use of implant parts for biomedical applications.

### 3.2. Biological Corrosion Resistance

**Figure 3:**
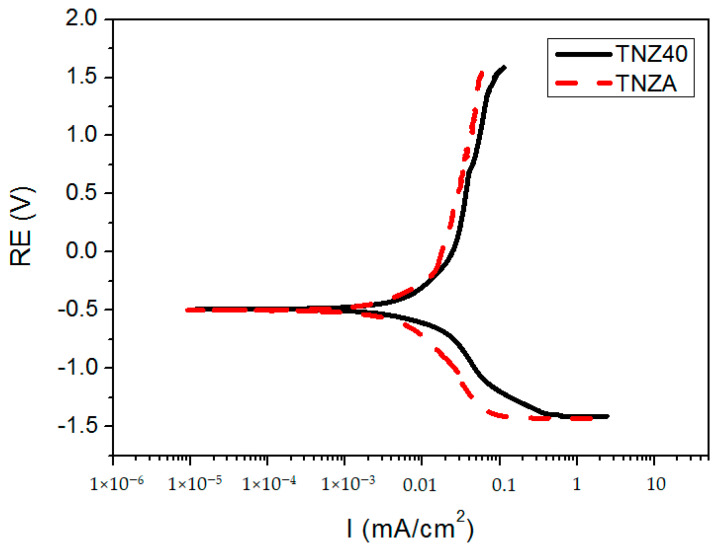
Tafel curves of Ti–39Nb–6Zr+0.45Al (TNZA) and Ti–39Nb–6Zr (TNZ40) in the 0.9% NaCl (pH = 7).

Figure 3 shows the results of the potentiodynamic polarization experiment using the TNZA and TNZ40 alloys in the 0.9% NaCl (pH = 7) solution, the normal saline solution. The corrosion current densities (*I_corr_*) of the TNZA and TNZ40 alloys were 2.104 and 3.157 μA/cm^2^, respectively, while their corrosion rates were 0.772 and 1.139 mpy, respectively. The increase in *I_corr_* implies that the current flows largely into the specimen, which leads to a high degree of corrosion. The corrosion resistance (*R_p_*) was obtained using the Stern–Geary equation,

(1) $$i_{corr}=\frac{\beta_{a}\beta_{c}}{2.303R_{p}\left(\beta_{a}\;+\;\beta_{c}\right)},$$

where *β_a_* and *β_c_* were obtained using the Tafel extrapolation method. The *R_p_* values of the TNZ40 and TNZA alloys obtained using this equation were 20.04 and 32.83 Ω/cm^2^, respectively. In addition, these two specimens consisted of stable passive films without pitting up to 1.5 V. The experimental values indicate that the corrosion resistance of the TNZA alloy is better than that of the TNZ40 alloy.

When a metal is inserted into the human body, the surface of the metal may produce corrosion products or release metal ions, which may cause problems in the human body. It is essential to determine the corrosion resistances of the alloys with Al as an alloy element because the elution of Al ions in vivo may cause neurotoxicity problems [20]. Alloys with Nb, added as an alloy element, exhibit corrosion properties similar to those of commercially pure titanium [21]. In addition, the alloys with Nb and Zr, added as alloy elements, consist of highly stable passive films because the oxide film of the surface is composed of a combination of oxides such as Nb_2_O_5_ and ZrO_2_ as well as TiO_2_ [22,23]. In vivo experiments with the Ti–15Zr–4Nb–4Ta alloy showed very small amounts of Ti, Zr, Nb, and Ta ions in the body [24]. In addition, Okazaki et al. reported no significant difference in the amount of Al ions in the body upon the insertion of implants composed of Ti–6Al–4V alloys into the body [25]. Therefore, the probability of neurotoxicity due to the elution of Al ions is very low because the corrosion resistance of the TNZA alloy is better than that of the TNZ40 alloy, and the Al content is considerably lower than that of the Ti–6Al–4V alloy.

### 3.3. In Vitro Properties

**Figure 4:**
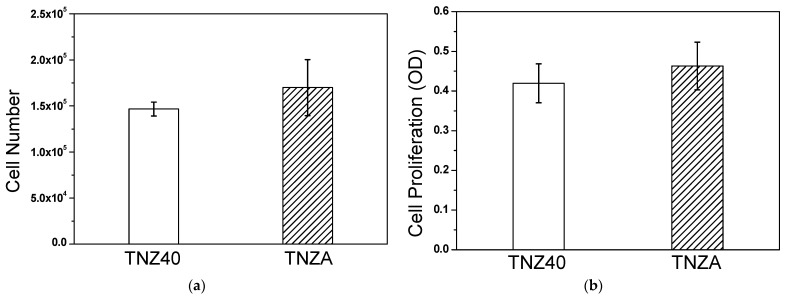
(**a**) Number of cells and (**b**) cell proliferation after 72 h.

When cells are dispensed on a specimen, it is considered that a higher proliferation of the cells implies a higher biocompatibility. In this experiment, 1.223 × 10^5^ cells were dispensed on the specimen. When the cells were placed on the specimen, the recovery rate was not 100%, because not all cells stick to the specimen. In addition, considering the doubling time of the MG-63 cells, only approximately 25–30% of the cells were initially attached to the specimen and proliferated. According to the counting of the cells after cultivation for 72 h, the cell proliferation rate of the TNZA alloy was similar to that of the TNZ40 alloy, assuming that the initial adhesion rates were equal (Figure 4a). The results of the absorbance measurement showed the same tendency as that of the results of the counting of the cells (Figure 4b), as summarized in Table 1.

### 3.4. In Vivo Properties

**Figure 5:**
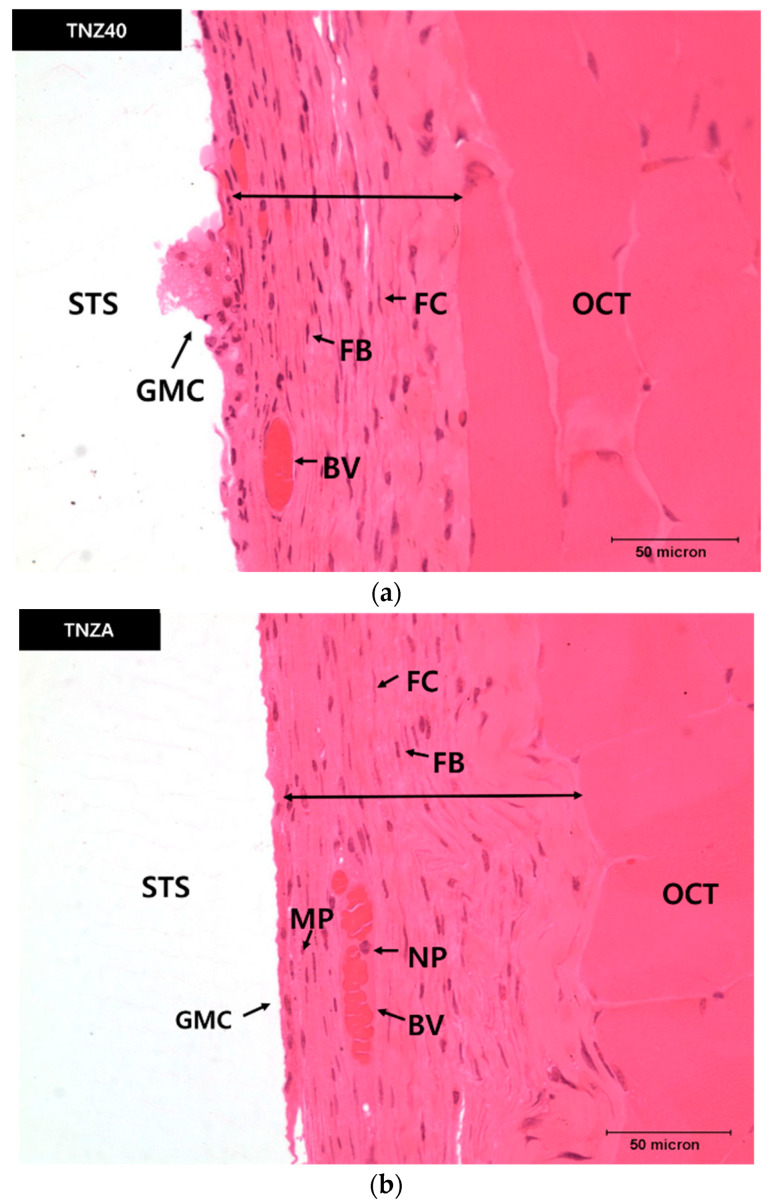
Photomicrographs of cells around the inserted titanium specimens: (**a**) TNZ40, (**b**) TNZA; FB: fibroblast, FC: fibrocyte, STS: space formerly occupied by the specimen, OCT: old connective tissue, MP: macrophage, NP: neutrophil.

**Figure 6:**
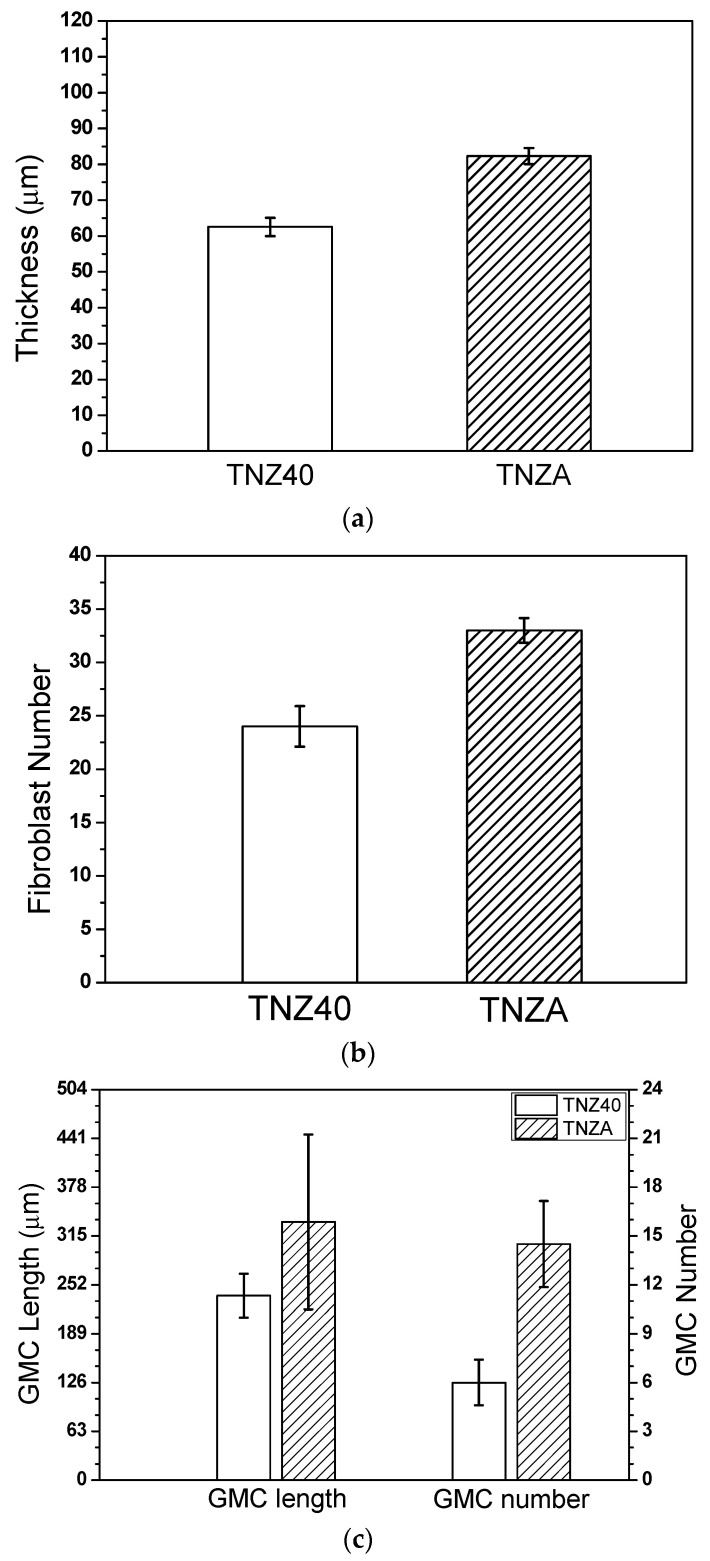
In vivo test results four weeks after the insertion of the titanium specimens: (**a**) thickness of the fibrous capsule around the specimen, (**b**) number of fibroblasts, and (**c**) length and number of giant multi-nucleated cells (GMCs).

The thickness of the newly created fibrous connective tissue around the specimen was measured after implanting the specimen into the abdominal connective tissue for four weeks to determine and compare the biocompatibilities of the TNZ40 and TNZA alloys. It is considered that a thinner fibrous tissue implies a higher biocompatibility [26,27]. Figure 5 shows the newly created fibrous connective tissue membranes and various cells, including fiber cells, macrophages, and neutrophils. The average thicknesses of the connective membranes of the mouse muscle tissues implanted with the TNZA and TNZ40 alloys were measured to be 82.3 µm and 62.5 µm, respectively (Figure 6a). According to the *t*-test, the thicknesses of the muscle tissue membranes exhibited a significant difference (*p* < 0.05). The number of fiber cells was additionally measured to ensure data reliability of the membrane thickness after measuring the thickness of the fibrous connective tissue membrane, as shown in Table 2. Fiber cells create a fibrous connective tissue membrane, which increases the thickness of the connective tissue membrane. According to the measurements of the numbers of cells, the TNZA and TNZ40 alloys exhibited 33 and 24 cells on average, respectively (Figure 6b). GMCs were observed in the entire newly created connective tissue membrane. GMCs are connected to the surface of the specimen and represent an array of nuclei, with a higher degree in the TNZA alloy than in the TNZ40 alloy. Fewer GMCs imply a higher biocompatibility because the GMCs lead to resistance to the specimen [28]. The average numbers of GMCs for the TNZA and TNZ40 alloys were 14 and 6, respectively. The average total lengths of GMCs for the TNZA and TNZ40 alloys were 333 and 238 µm, respectively (Figure 6c). However, no reactions of the tissues such as necrosis and bleeding around the specimens with the TNZ40 and TNZA alloys were observed. The in vivo experiment showed that these two alloys exhibited excellent biocompatibilities. The in vivo properties of the TNZA alloy were slightly weaker than those of the TNZ40 alloy.

Blood vessels (BVs) were observed in the newly created connective tissue membranes. Some inflammatory cells, such as macrophages and neutrophils, were also observed. BVs participate in membrane thickening by facilitating the oxygen supply. Some empty BVs were also observed. A surgical treatment of inserting a specimen into an animal body may lead to biologic reactions in tissue degeneration, such as bleeding, necrosis, and discoloration. However, this was not observed in this experiment.

## 4. Conclusions

In this study, the feasibility of the implant materials for biomedical applications was investigated by analyzing the improved mechanical, chemical, and biocompatibility properties of the TNZA alloy, which was obtained by adding a small amount of Al to the TNZ40 alloy.

1. The YS was improved by approximately 20% owing to the deformation of the lattices by the formation of a substitutional solid solution and resulting obstruction of the dislocation movement by the Al alloying element added to the TNZ40 alloy. Thus, it can be utilized as a biocompatible implant material.
2. The *I*_corr_ values of the TNZA and TNZ40 alloys were 2.104 and 3.157 μA/cm^2^, respectively. Pitting was not observed, because these two alloys consisted of stable passive films up to 1.5 V. This confirmed that the corrosion resistance of the TNZA alloy was better than that of the TNZ40 alloy and that the elution of Al ions does not lead to problems.
3. The degrees of cell proliferation and absorbance were similar to those of the TNZ40 alloy, according to the evaluation of the biocompatibility of the TNZA alloy through the in vitro experiments (cell proliferation and measurement of the absorbance). According to the in vitro experiments, no harmful effects of the addition of a small amount of Al were observed.
4. According to the measurement of the thicknesses of the newly created fibrous connective tissue membranes four weeks after inserting the specimens of TNZA and TNZ40 into the mouse abdominal subcutaneous tissues, although the membrane thickness, number of fiber cells, and number and length of the GMCs of the TNZ40 alloy were smaller than those of the TNZA alloy, the differences were not significant. In addition, no tissue reactions such as tissue necrosis and bleeding around the inserted specimen were observed.

## Figures and Tables

**Table 1:** In vitro test results of cell proliferation experiment.

Specimens	Cell Number (×10^3^)	Cell Proliferation (×10^−1^)
TNZ40	146.7 ± 7.6	4.19 ± 0.49
TNZA	170.0 ± 30.4	4.63 ± 0.6

**Table 2:** Comparison of various biocompatibility characteristics after insertion of TNZA and TNZ40 specimens into mice.

Specimens	Thickness (μm)	Fibroblast Number	GMC Length (μm)	GMC Number
TNZ40	62.5 ± 2.6	24 ± 1.9	238 ± 28.2	6 ± 1.4
TNZA	82.3 ± 2.2	33 ± 1.2	333 ± 113	14 ± 2.6

## Data Availability

The data presented in this study are available on request from the corresponding author.
